# Fermented calcium butyrate supplementation in post-peak laying hens improved ovarian function and tibia quality through the “gut-bone” axis

**DOI:** 10.1016/j.aninu.2023.10.008

**Published:** 2024-01-03

**Authors:** Huaiyong Zhang, Yongshuai Wang, Yilu Wang, Bin Wei, Leilei Wang, Minh Tu Nguyen, Xiangyun Lv, Yanqun Huang, Wen Chen

**Affiliations:** a College of Animal Science and Technology, Key Laboratory of Animal Biochemistry and Nutrition, Ministry of Agriculture, Henan Agricultural University, Zhengzhou, 450046, China; b Laboratory for Animal Nutrition and Animal Product Quality, Department of Animal Sciences and Aquatic Ecology, Ghent University, Ghent, 9000, Belgium; c Department of Agriculture and Forestry, Hue University, Hue, 49000, Viet Nam; d Charoen Pokphand Group Co., Ltd. Zhumadian, 463000, China

**Keywords:** Fermented Ca butyrate, Ovarian property, Tibia metabolism, Layer

## Abstract

The compromised egg quality and leg abnormality during the end of the laying cycle (after 40 weeks) have been leading to poor animal welfare and substantial economic losses. Therefore, the effects of fermented calcium (Ca) butyrate, produced by fermentation by *Clostridium butyricum*, on production, eggshell quality, and tibial property of hens were explored. A total of 192 Hy-line brown laying hens at 50-week-old were assigned to a basal diet or the basal diet with 300 mg/kg of the fermented Ca butyrate from 50 to 58 weeks of age. Each treatment had 6 replicates with 16 hens each. The diet supplemented with 300 mg/kg fermented Ca butyrate notably increased egg weight, ovarian follicle number, and eggshell strength (*P* = 0.072) as compared to the basal diet, which were associated with cytokine secretion, toll-like receptor signaling pathways, and intestinal immunity based on the RNA-seq data from the granulosa. Dietary Ca butyrate inclusion decreased the expression of ileal tumor necrosis factor-alpha and serum pro-inflammatory cytokine concentration, as well as increased the content of serum immunoglobulin A when compared to the basal diet (both *P* < 0.05). The birds that received fermented Ca butyrate diets exhibited higher villus height (*P* < 0.05) and upregulated expression of tight junction proteins, whereas it did not alter the composition of cecal microbiota (*P* > 0.05). In addition, the diet with fermented Ca butyrate reduced the number of osteoclasts in the proximal tibia and the level of C-terminal cross-linked telopeptide of type I collagen, a bone resorption marker (*P* < 0.05), whereas it tended to increase the concentration of the procollagen type I N-terminal propeptide that reflects bone formation marker in serum. Moreover, the layers fed fermented Ca butyrate diets possessed higher (*P* < 0.05) bone area and trabecular number of the proximal tibia, yield load, and ultimate load than those that consumed basal diets. Collectively, dietary fermented Ca butyrate supplementation in post-peak layer diets improved the ovarian function and tibia quality, which might be related to enhancing intestinal integrity and consequently decreasing inflammation mediated bone resorption.

## 1 Introduction

The decrease in both internal and external egg quality such as eggshell quality has been widely observed as laying age increases in laying hens (Jiang et al., 2014; Samiullah et al., 2017), especially when the layer is older than 40 weeks (Bain et al., 2016). These alterations are closely linked to the influences of chemokines, pro- and anti-inflammatory cytokines during the ovulatory cycle. It was noticed that the expression of inflammatory cytokines (Elhamouly et al., 2018a) and the number of macrophages tends to increase with age in the oviduct of laying hens (Zheng and Yoshimura, 1999). Moreover, interleukin (IL)-1β and IL-6 have been assumed to affect eggshell biomineralization by interfering with the expression of calcium (Ca) binding protein (Nii et al., 2018). Furthermore, the immune system is also improved to affect the inflammatory status in the ovary through the high frequency of ovulation rate and the spread of microbial infection which accompanies leukocyte infiltration and the production of pro-inflammatory cytokines (Zhong et al., 2016). In addition, compromised eggshell quality might result from the incidence of physical bone abnormalities in hens. In this regard, bone, particularly long bone, in egg-laying hens is comprised of cortical, trabecular, and medullary bone tissues, which is responsible for supporting body weight and storing mineral for egg-shell formation, respectively. During the laying cycle, the bone strength provided by cortical and trabecular bone tissues will gradually decrease as a result of insufficient bone formation and/or excessive bone resorption, whereas medullary bone can gradually accumulate for supporting eggshell formation (Dacke et al., 1993). A negative correlation has been observed among egg production, eggshell thickness, and bone breaking strength (Kim et al., 2005; Leyendecker et al., 2001). Furthermore, laying hens selected for egg production are more susceptible to osteoporosis owing to a negative Ca balance, which is due to the high demand for Ca during eggshell formation (Whitehead and Fleming, 2000). Therefore, improving egg production and quality in post-peak layer hens might be dependent upon attenuating ovarian inflammation and optimizing bone quality.

Although direct evidence cannot be found in terms of the relationship among the gut microbiota, intestinal barrier, and ovarian health, the improved production and egg quality due to enhanced intestinal morphology, integrity, and/or microbial composition in laying hens (Abbasi et al., 2021; Gan et al., 2020; Zhang et al., 2022c) could be attributed to the reduction in the inflammatory response of ovary. Indeed, in addition to its function in the absorption and metabolization of nutrients, the intestine also plays a crucial role in the immune system as a physical barrier by preventing the passage of pathogens, antigens, and bacterial toxins into the systemic circulation and secreting immunological molecules. What is noteworthy is the interaction of the intestinal barrier and inflammation in bone metabolism. Impairing intestinal epithelial integrity challenged with *Salmonella* infection (Raehtz et al., 2018) or heat stress (Zhang et al., 2021) led to lower density of trabecular bone in chickens, which might have been attributed to increased systemic inflammation, eliciting osteoclastic bone resorption (D'Amelio and Sassi, 2018). Moreover, a decreased level of osteoclasts, IL-6, and tumor necrosis factor-alpha (TNF-α) in osseous tissue was observed in germ-free mice which presented a higher bone mass when compared with conventionally raised mice (Sjögren et al., 2012). Outcomes from our recent study also demonstrated that improvement in intestinal integrity could decrease systemic inflammation and bone resorption, and consequently improve tibial properties in broilers (Zhang et al., 2021, 2022a) and laying hens (Zhang et al., 2022b). This is the so-called “gut-bone” axis. This evidence probably explains the reasons why a lot of effort has been made in the last few decades to regulate “gut-bone” axis and increase the bone mass of laying hens through the supplementation of organic acids (Kazempour and Jahanian, 2017; Zhang et al., 2022c), probiotics (Wang et al., 2021), essential oils (Zhang et al., 2022b).

Among these organic acids, butyric acid, a major metabolic by-product of *Clostridium butyricum* (*C. butyricum*), *Butyrivibrio crossotus*, *Roseburia*, etc., was found to play a vital role in promoting intestinal integrity as it is the primary energy source of intestinal epithelial cells and stimulates both the proliferation and differentiation of enterocytes (Abdelqader and Al-Fataftah, 2016). Besides, this fatty acid also has antimicrobial properties, acting as an anti-inflammatory agent and assisting in intestinal motility (Celasco et al., 2014). In practice, considering the strong unpleasant odor of butyric acid, the salt form of butyric acid is preferred in animal nutrition such as sodium butyrate and Ca butyrate (Chamba et al., 2014). Once fed to birds, sodium butyrate can be converted to butyric acid within the avian alimentary canal and positively affect avian intestinal health and physiological activities (Elnesr et al., 2019). It was reported that dietary supplementation with sodium butyrate could enhance intestinal morphology and performance in broilers (Kaczmarek et al., 2016), as well as improve the yolk color and microbial diversity in a dose-dependent manner in laying hens (Zhang et al., 2022c). Similarly, Ca butyrate, functioning as butyric acid in the acidic environment of the proximal digestive tract of birds, was also noted to improve the growth performance, intestinal health, and carcass traits in broilers (Gümüş et al., 2021; Ocejo et al., 2017). Further to this, it also increased body weight gain and exhibited anti-inflammatory/antioxidant effects in Japanese quails (Abd El-Wahab et al., 2019), and improved eggshell quality in aged laying hens (Song et al., 2022). Moreover, *C. butyricum* belongs to a butyric acid-producing Gram-positive anaerobe and principally colonizes the distal small intestine and colon of animals. The *C. butyricum* supplementation in the diet of broiler could promote growth performance, intestinal morphology, and ameliorate inflammation (Takahashi et al., 2018; Yang et al., 2012), as well as manipulate gut microbiota by increasing the abundance of some beneficial bacteria and decreasing the proportion of certain harmful bacteria (Zhang et al., 2014). Dietary supplementation with 1 × 10^9^ CFU/kg of *C. butyricum* in yellow-feathered breeder hens from 45 to 54 weeks of age significantly increased the laying rate, eggshell thickness, and jejunal morphology, along with remarkably decreasing the IL-6 level in the jejunum (Wang et al., 2021). Supplemental *C. butyricum* could also shape the gut microbiota of aged laying hens, demonstrated by an increased Bacteroidetes proportion and decreased Firmicutes abundance in the ileum (Wang et al., 2020). These alterations in intestinal integrity, gut microbiota, and inflammatory cytokines due to the administration of Ca butyrate and/or *C. butyricum* have been proven exert a key role in laying performance (Song et al., 2022; Wang et al., 2021) and bone metabolism (D'Amelio and Sassi, 2018). Hereby, the production of fermented Ca butyrate from biomass using *C. butyricum* (fermented Ca butyrate) probably enhances the ovarian function and bone quality of post-peak layer hens.

This study, therefore, aimed to explore if supplemental fermented Ca butyrate could be a viable approach for improving production performance, eggshell strength, and tibia quality of laying hens in the late stage of production. Also, the mechanisms underlining the relationship between fermented Ca butyrate and ovarian function, or the “gut-bone” axis were investigated using transcriptome sequencing (RNA-seq) and 16S rRNA sequencing. This study provides a new understanding and a potential means by which to extend the laying period of hens and decrease the incidence of leg problems for aged laying hens.

## 2 Materials and methods

### 2.1 Animal ethics statement

Care, handling, and sampling procedures were approved by the Animal Care and Use Committee of Henan Agricultural University before initiation of the trial (HNND-2021-004).

### 2.2 Fermented Ca butyrate preparation

Fermented Ca butyrate used in the current study was provided by Charoen Pokphand Group Co., Ltd. (Henan, China). In detail, the fed-batch fermentations of *C. butyricum* (CP-BIO 3000), a strain that comes from the Bio-Business Line of Charoen Pokphand Group, were performed in a 5-L stirred-tank bioreactor containing 2 L of the medium with glucose as the substrate. The fermentation medium was continuously stirred at 100 rpm and sparged with nitrogen to maintain anaerobiosis. Fermentations were carried out at 37 °C and pH 7.0 ± 0.1 controlled by adding calcium carbonate (CaCO_3_) based on the dissociated state of Ca butyrate for 3 d. At the end of fermentation, the supernatants were collected for Ca butyrate determination through ethylene diamine tetraacetic acid (EDTA) titration and the analysis of butyrate using high-performance liquid chromatography (HPLC; Agilent 1100 series, Agilent, USA), and the results showed that the content of Ca butyrate was 50% to 52%. Finally, the fermentation broth was dried by spray-drying stored at 4 °C until utilization.

### 2.3 Experimental design

In this study, a total of 256 Hy-line brown laying hens were pre-fed a corn-soybean meal basal diet for 2 weeks to adapt to the environment, and then the birds with similar egg production rates (192 in total) were selected and divided at random into the basal diet group (Ctrl group) and the basal diet with 300 mg/kg of the fermented Ca butyrate group (Ca butyrate group) using a randomized block experiment design, in which each treatment included 6 replicates of 16 hens each. The number of replicates per treatment was established based on statistical power analysis using G∗Power 3 software (Franz Faul, Christian-Albrechts-Universität Kiel, Kiel, Germany). The experimental period lasted from 50 to 58 weeks of age. The basal diet was formulated to meet the recommendations for laying hens of the China National Feeding Standard (NY/T 33-2004) with 3.6% of Ca and 0.3% of available phosphorus (P), where the Ctrl group, received a layer diet containing fine limestone particles as the Ca source (Table S1). To avoid the effects of feed processing on fermented Ca butyrate, the diet was supplemented in powder form. All hens were housed in 4-layer vertical cages located in a temperature-controlled room with a lighting schedule of 16 h of light and 8 h of darkness. The ambient temperature was 23 to 25 °C with 30% to 50% relative humidity. Feed and water were provided ad libitum.

### 2.4 Data collection and chemical analysis

The birds were weighed in the 50th and 58th weeks. Feed intake was recorded weekly on a replicate basis. The egg production and egg weight were recorded daily, and then the laying rate, average daily feed intake (ADFI), egg mass, and feed conversion ratio were calculated from 50 to 58 weeks. Here, the egg mass was expressed as the laying rate (%) multiplied by the average weight of eggs (g), and the feed conversion ratio was determined as the ratio of total feed intake (g) to the total egg weight (g).

To measure apparent metabolism energy and nutrients in diets, the clean, fresh, feather- and feed-free excreta from each cage were collected using trays under the cages for continuous 3 d, homogenized, dried at 60 °C for 3 d, and ground. The diets and excreta samples were analyzed for dry matter (method 934.01) and gross energy using a bomb calorimeter (Parr Instrument, Moline, IL, USA) using standard procedures of Association of Official Analytical Chemists (AOAC, 2006), as well as acid-insoluble ash as a marker for digestibility according to the previous method (Spranghers et al., 2018). The crude protein content in the diets defined as nitrogen multiplied by 6.25 was determined based on method 990.93 (AOAC, 2006). In addition, the Ca and P contents of feeds were determined through EDTA and ammonium metavanadate colorimetry, respectively. The results of dietary nutrient analysis confirmed proper preparation of experimental diets (Table S2).

### 2.5 Sampling

On the last day of the trial, 1 bird from each cage was randomly selected for blood collection from the brachial vein to obtain serum. After sacrificing by cervical dislocation, about 1 cm of mid-ileum and left proximal tibia were dissected for histomorphological analysis. The mid-ileal mucosa (removing the histomorphological sample) and cecal contents were collected for gene expression and microbiome sequencing, respectively. The right tibia was obtained for bone growth and mineralization determination. The ovaries were removed, and the number of follicles with over 4 mm diameter were counted. Subsequently, the pre-grade white follicles (diameter of 1 to 6 mm) were collected for RNA-seq analysis. At 58 weeks of age, 24 eggs from each treatment were collected to determine eggshell thickness and strength using a strength meter (EFG-0503, Robotmation Co., Ltd. Japan).

### 2.6 RNA-seq analysis

The granulosa cells were separated from pre-grade white follicles (diameter of 1 to 6 mm) for RNA-seq as previously described (Kovács et al., 1992). Briefly, the follicle was inverted to peel off the granulosa layer, and all follicle layers were subjected to trypsin dispersion (1 mg/mL) followed by layering onto a Percoll suspension (50%) to remove debris and red blood cells. Total RNA was extracted from isolated granulosa cells. After determining the quantity and quality of the extracted RNA, a SMART-Seq Ultra Low Input RNA kit (Takara Bio Inc., Kusatsu, Japan) was used to generate amplified cDNA from 500 ng of starting RNA. Subsequently, sequencing libraries were generated using the TruSeq RNA Sample Preparation Kit (Illumina, San Diego, CA, USA) prior to sequencing on an Illumina NovaSeq 6000 platform (Novogene Biotechnology Co., Ltd, Beijing, China). Low-quality sequences (phred < 20) were removed from the raw data. Reads were mapped to the reference genome (GRCg6a) by using HISAT2 software. The differentially expressed genes (DEG) between Ctrl and Ca butyrate groups were analyzed by the HISAT2 software. Genes with log2|FC| ≥ 1 and *P* < 0.05 were considered as significant DEG. Gene Set Enrichment Analysis (GSEA) was performed using gsea2-2.2.3 in R software. Kyoto Encyclopedia of Genes and Genomes (KEGG) analysis of DEGs was performed via the database for annotation, visualization, and integrated discovery (DAVID) (https://david.ncifcrf.gov). Sequencing files were deposited to the BioProject database in NCBI (accession numbers PRJNA947743).

### 2.7 Serum inflammation and bone turnover

Commercial kits (Meimian Industrial Co., Ltd., Jiangsu, China) were applied for the concentrations of serum TNF-α, IL-1β, IL-6, IL-10, and transforming growth factor beta (TGF-β). The catalog numbers of the kits were MM-093802, MM-3691002, MM-052102, MM-114502, and MM-228702, respectively. Serum immunoglobulins (Ig) including IgG, IgA and IgM were analyzed by the commercial chicken-specific enzyme-linked immunosorbent assay (ELISA) kits using diluted serum samples (1:125,000 for IgG; 1:10,000 for both IgA and IgM). Bone turnover markers in serum including alkaline phosphatase (ALP), procollagen type I N-terminal propeptide (P1NP), and C-terminal cross-linked telopeptide of type I collagen (CTx) were quantified with commercial assay kits (MM-261602, MM-0923R2, and MM-194802 for ALP, P1NP, and CTx, respectively). All kits were obtained from Meimian Industrial Co., Ltd. (Jiangsu, China). All samples were tested in triplicate within each assay in accordance with the manufacturer's instructions. Serum Ca and P content were measured through the chromogenic complex with o-cresol phthalein and the molybdenum blue method using a Bio-chemistry Analyzer (Yellow Springs Instrument Co. Inc., Yellow Springs, OH, USA), respectively.

### 2.8 Gene expression by real-time PCR

Total RNA was isolated from the mid-ileal mucosa and granulosa cells using a total RNA extraction kit (TransGen Biotech Co. Ltd, Beijing, China) in accordance with the manufacturer's instructions. The quality and concentration of extracted RNA were determined by agarose gel electrophoresis and nucleic acid quantification, respectively. The cDNA was synthesized by the cDNA reverse transcription kit (Takara, Dalian, China). The obtained cDNA was used for gene expression analysis using SYBR green qPCR master mix (Takara, Dalian, China). The amplification conditions are as follows: 95 °C for 15 s followed by 40 cycles of 95 °C for 30 s and 60 °C for 34 s with a final melting curve analysis. The specific primers were designed online (https://bioinfo.ut.ee/primer3-0.4.0/) and showed in Table S3. The β-actin was used as a housekeeping gene to normalize target gene expression.

### 2.9 16S rRNA sequencing of cecal microbiota

Total genome DNA was extracted using a Stool DNA Isolation Kit (Norgen Biotek Corp., Thorold, Canada). After determination of DNA content and purity, amplification of the V4 region of the 16S rRNA gene was achieved by using specific primers 515F (5′-GTGCCAGCMGCCGCGGTAA-3′) and 806R (5′-GGACTACHVGGGTWTCTAAT-3′). Then, sequencing was performed using the Illumina HiSeq platform (Novogene Biotech Co., Ltd., Beijing, China). Clusters of high-quality sequences have been categorized as operational taxonomic units (OTU) at a 97% identity threshold based on the UPARSE algorithm in USEARCH (v7.0.1090). Taxonomy was assigned using the SILVA database (v1.32). STAMP with *t*-tests was used to examine the differences in microbial abundances. The alpha diversity was evaluated with MOTHUR by calculating the Shannon and Simpson index using the OTU table in R. Beta-diversity between Ctrl and Ca butyrate groups was visualized using a principal coordinate analysis (PCoA) based on Bray–Curtis distance. Sequences generated in the current study have been deposited in the NCBI database under the accession number PRJNA949874.

### 2.10 Ileum histological analysis

The mid-ileum was fixed in 10% buffered formaldehyde, dehydrated, embedded in paraffin, and sliced (5-μm thickness). After staining with hematoxylin-eosin (H&E), the villus height and crypt depth of at least 10 well-oriented villi were measured and the ratio of villus height to crypt depth was calculated as the previous description (Zhang et al., 2021).

### 2.11 Bone staining and histological analysis

The fixed proximal end of the tibia was decalcified with 10% EDTA (pH 7.4), embedded, and sliced. Subsequently, the section was de-paraffinized, rehydrated, and stained with tartrate-resistant acid phosphatase (TRAP) and toluidine blue staining solution (Sigma–Aldrich, Inc., MO, USA), respectively. Histopathological images were collected using a microscope equipped (Nikon Corporation, Tokyo, Japan). Undergoing the grayscale transform, the toluidine blue slides were used for the histological analysis as previously described (Vidal et al., 2012), i.e., total area (T.Ar), calcified bone area (B.Ar), bone perimeter (B.Pm) can be obtained directly from images, and then the trabecular bone area (Tb.Ar), number (Tb.N), and thickness (Tb.Th) were calculated as the following equations.

Tb.Ar(%)=B.Ar/T.Ar×100,

Tb.Th(μm)=2×B.Ar/B.Pm,

Tb.N(No./mm)=(B.Ar/T.Ar)/Tb.Th.

### 2.12 Bone dimension, strength, and mineral content

The tibial length and circumference of the middle part of the tibia were measured by vernier calipers and flexible rulers, respectively. These tibias were then weighed after being dried by filter paper, and the relative tibia fresh weights were calculated as the ratio of fresh weight to body weight. Subsequently, biomechanical testing was performed by the 3-point bending method with a texture analyzer (TA-XT Plus. Stable Micro Systems, Surrey, UK) at a constant 50 kg load cell. Loading proceeded at the mid-point of the tibia at a constant rate (5 mm/min) up to the breaking of the bone. Force-displacement data were collected, and the whole bone stiffness, yield load, and ultimate load were calculated, in which the stiffness was defined as the slope of the linear portion of the load–displacement curve, and the yield point was considered as the load at which the load-deformation relationship ceased to be linear (Turner, 2006). In addition, the mineral content indicated by ash, Ca, and P was determined based on the method previously described (Zhang et al., 2019). Briefly, the tibia was defeated by immersing in ethyl ether, air-dried for 24 h at room temperature, and ashed in a muffle furnace at 550 °C for 24 h. The ash content was calculated based on the percentage of dry-defatted weight. Ca and P contents (% dry-defatted weight) in ash were determined through EDTA titration and ammonium metavanadate colorimetry, respectively.

### 2.13 Statistical analysis

Each pen was used as the experimental unit. The statistical power of least 0.78 (78%) was obtained based on the significance level of 0.05 and the effect size, which was calculated through mean and standard deviation using G∗Power 3 software (Franz Faul, Christian-Albrechts-Universität Kiel, Kiel, Germany). The data obtained were subjected to the Shapiro–Wilk and Levene's test for normal distribution and homogeneity of variances, respectively. When normal distribution was confirmed, the comparisons were performed with using a two-tailed unpaired *t*-test using the following statistical model:

$$Y_{j}=\mu +D_{j}+\varepsilon_{j},$$

where *Y*_*j*_ the mean value of treatment *j* (Ctrl and Ca butyrate), μ is the overall mean, *Dj* is the fixed effect of treatment *j*, and $\varepsilon_{j}$ is the error term. Block was further included as random factor if significant. When *P* < 0.05 and *P* < 0.01, the variation was considered statistically significant and of tendency, respectively. Data is reported as mean ± standard deviation.

## 3 Results

### 3.1 Production performance and ovarian follicle numbers

**Fig. 1:**
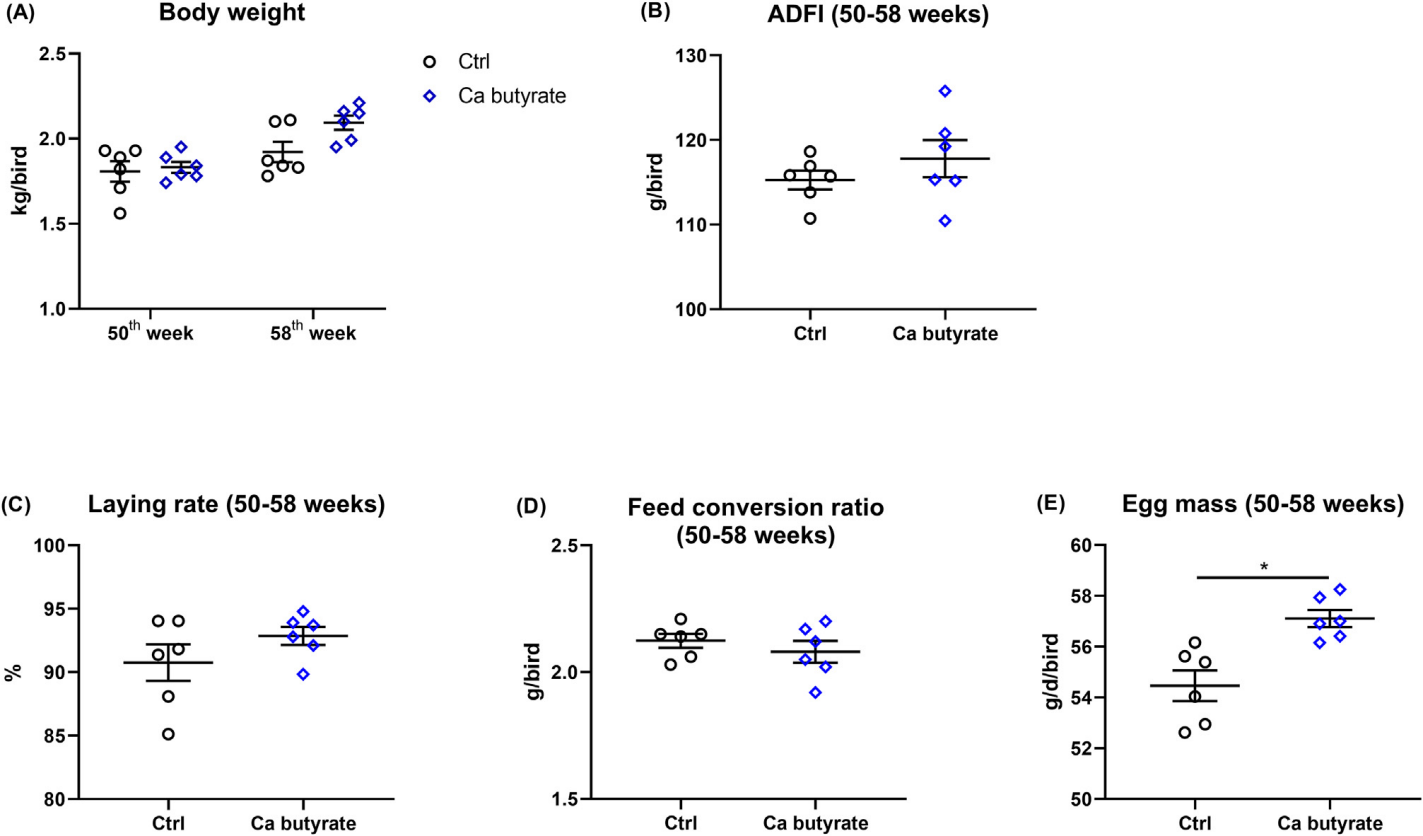
Fermented calcium (Ca) butyrate effect on laying performance including (A) body weight, (B) average daily feed intake (ADFI), (C) laying rate, (D) feed conversion ratio, and (E) egg mass, in which the feed conversion ratio was determined as the ratio of total feed intake (g) to the total egg weight (g), and the egg mass was calculated by multiplying the laying rate (%). Data shown are means and standard deviation (*n* = 6), the point represents the mean of each replicate. ∗*P* < 0.05.

**Fig. 2:**
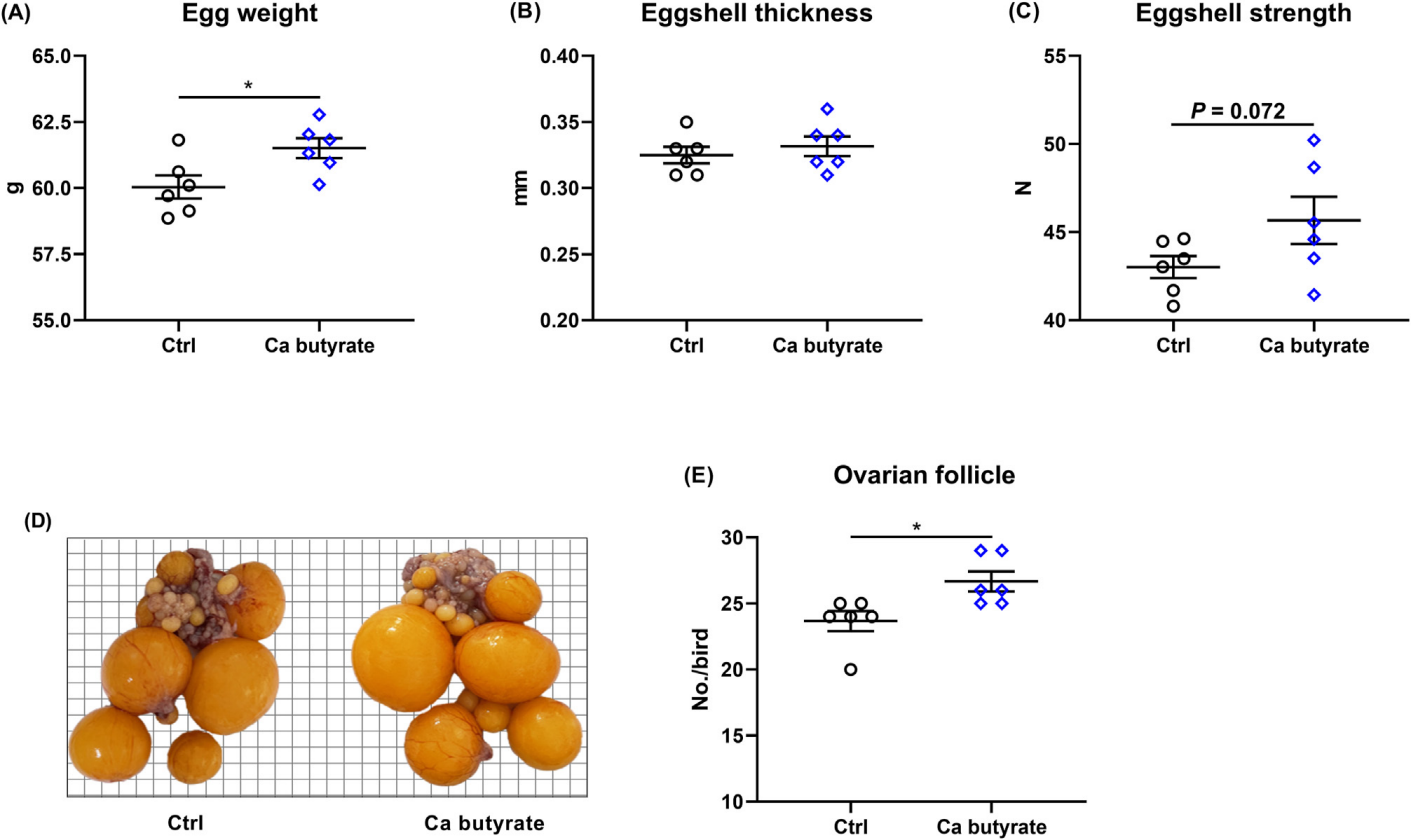
Dietary fermented calcium (Ca) butyrate treatment increases egg weight and ovarian follicle number of 58-week-old laying hens. (A) Egg weight, (B) eggshell thickness, (C) eggshell strength, (D) representative image of ovarian follicles, and (E) the number of ovarian follicles. Data shown are means and standard deviation (*n* = 6). ∗*P* < 0.05.

Supplementing 300 mg/kg fermented Ca butyrate in the diet of laying hens did not alter the body weight, ADFI, laying rate, and feed conversion ratio from 50 to 58 weeks in comparison to the Ctrl group (Fig. 1A–D), whereas the hens fed with fermented Ca butyrate supplemented diets demonstrated greater average egg mass (*P* < 0.05), as presented in Fig. 1E. In addition, at 58 weeks of age, when compared to Ctrl birds, the hens fed with Ca butyrate diets possessed higher egg weight (*P* < 0.05) and eggshell strength (*P* = 0.072), along with a similar thickness of eggshell (Fig. 2A–C). Of note, the number of ovarian follicles of 58-week-old laying hens was significantly (*P* < 0.05) higher in the Ca butyrate group than those of Ctrl group (Fig. 2D and E).

### 3.2 Differential transcriptome analysis

**Fig. 3:**
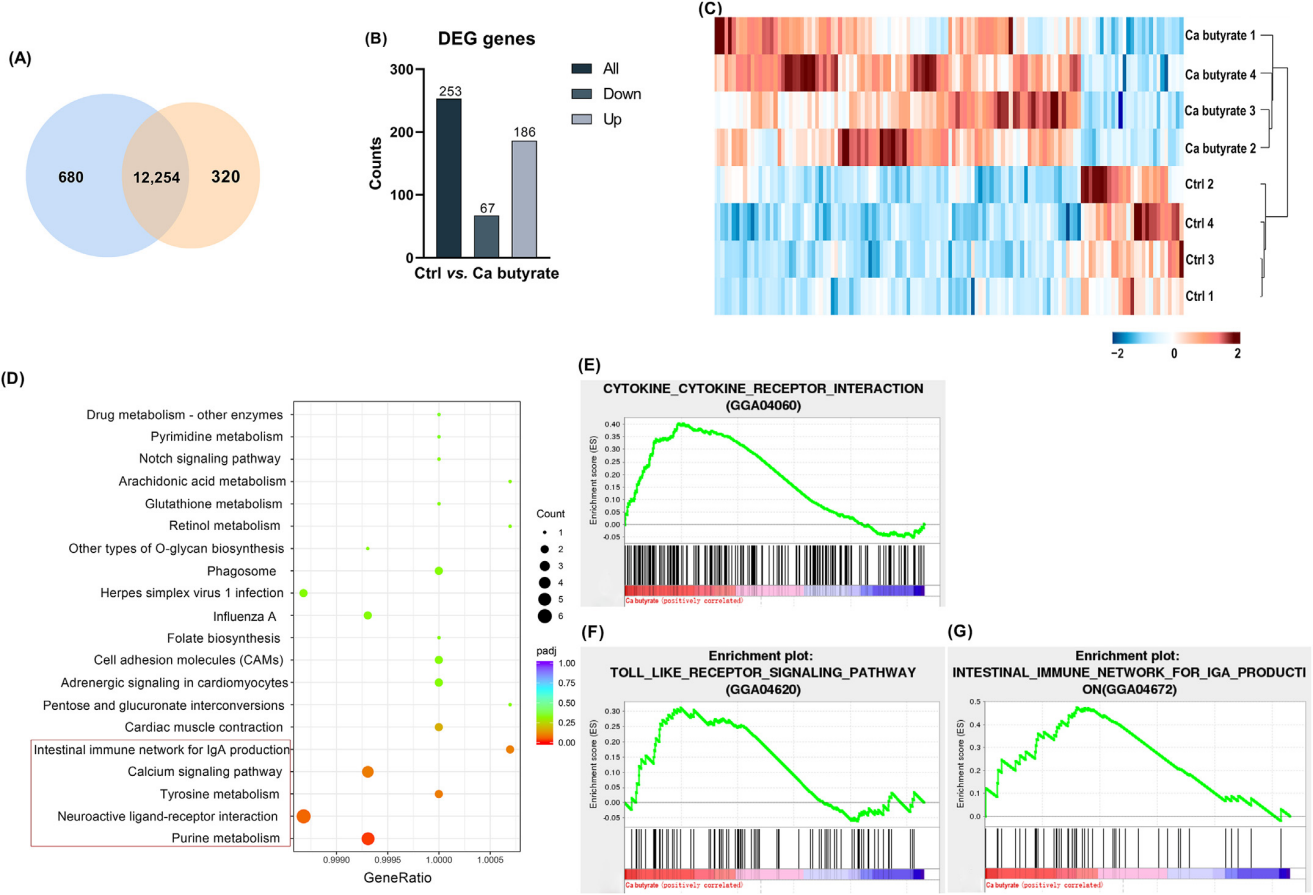
Transcriptome analysis for differentially expressed genes (DEG) after the dietary supplementation of fermented calcium (Ca) butyrate. (A) Venn diagram showing the distinct and overlapping genes of the transcriptome. (B) The quantity statistics of DEG. (C) Heatmap with hierarchical clustering analysis of DEGs of the transcriptome. The color scale indicates the mRNA expression levels. (D) Kyoto Encyclopedia of Genes and Genomes (KEGG)-enrichment analysis of differentially expressed genes. (E-G) Gene Set Enrichment Analysis (GSEA) analysis of the whole transcriptome. Genes with log2|FC| ≥ 1 and *P* < 0.05 were considered as significant DEG (*n* = 4).

To evaluate the effect of fermented Ca butyrate on ovarian function and related mechanisms, the granulosa cells from the Ctrl and Ca butyrate group were acquired for RNA-seq and RT-qPCR analysis. According to Fig. S1, the expression of anaplastic lymphoma kinase (*ALK*), prostaglandin D2 synthase (*PTGDS*), myosin XVA (*MYO15A*), tyrosine hydroxylase (*TH*), and osteocalcin-like protein OC3 (*OC3*) was consistent between the transcriptome data and RT-qPCR results, which confirmed the consistency and accuracy of the transcriptome data. Based on RNA-seq data, transcriptional gene expression was compared between the Ctrl and fermented Ca butyrate groups. In total, 12,254 genes were expressed in both the Ctrl and Ca butyrate groups, 680 and 320 genes were identified in the Ctrl and Ca butyrate groups, respectively (Fig. 3A). One hundred eighty-six up-regulated and 67 down-regulated DEG of transcriptome were identified based on log2|FC| > 1 and *P* < 0.05 (Fig. 3B). The heatmap data showed that the top 124 DEGs could significantly distinguish between the 2 groups of tissues (Fig. 3C). KEGG analysis identified significant enrichment pathways including purine metabolism, tyrosine metabolism, Ca signaling pathway, and intestinal immune network for IgA production (Fig. 3D). In addition, the outcomes of GSEA showed that several positive correlations were identified including cytokine, toll-like receptors (TLR) signaling pathways and intestinal immune pathway (Fig. 3E–G).

### 3.3 Ileal development and integrity

**Fig. 4:**
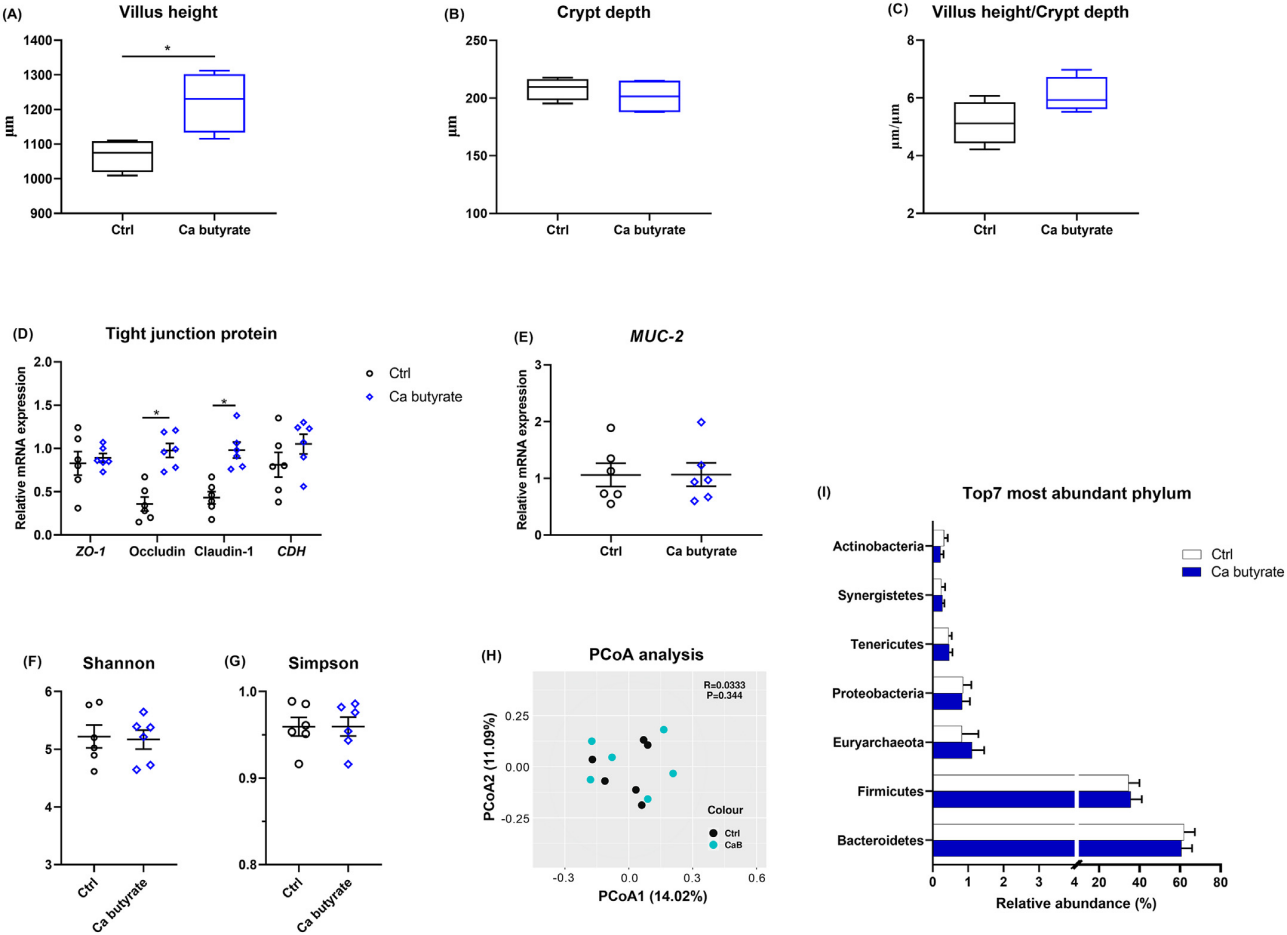
Ileal development and integrity, as well as cecal microbiota response to dietary calcium (Ca) butyrate supplementation. The mid-ileum was stained with hematoxylin/eosin staining, and then the (A) villus height, (B) crypt depth, and (C) the ratio of villus height to crypt depth were obtained based on the section (magnification, 100×), in which boxes are bounded by the 25th and 75th percentiles, with the median shown by the line bisecting the box. (D) The mRNA abundance of zonula occludens-1 (*ZO-1*), occludin, claudin-1, cadherin 1 (*CDH1*), and (E) mucin-2 (*MUC-2*) was determined for evaluating the integrity of the ileum. In addition, (F) Shannon index, (G) Simpson index, and (H) principal coordinate analysis (PCoA) based on Bray–Curtis dissimilarities were used to assess cecal microbiome diversity. (I) Relative abundances of the top 7 most bacteria at phylum level. Data shown are means and standard deviation (*n* = 6). ∗*P* < 0.05.

The influence of fermented Ca butyrate on ileal development is shown in Fig. 4. Dietary fermented Ca butyrate addition increased villus height (*P* < 0.05), whereas it did not change the crypt depth and villus height-to-crypt depth ratio in the ileum as compared to the Ctrl diet (Fig. 4A–C). Regarding the intestinal barrier, when compared to the Ctrl group, birds fed the Ca butyrate diet displayed significantly upregulated mRNA levels of occludin and claudin-1 (*P* < 0.05), and comparable transcript abundance of zonula occludens-1 (*ZO-1*), cadherin 1 (*CDH1*), and mucin-2 (*MUC-2*) (Fig. 4D and E).

### 3.4 Cecal microbiota

As illustrated in Fig. 4F–I, fermented Ca butyrate supplementation did not affect the alpha diversity indices (Shannon and Simpson). PCoA analysis was performed and showed that the first principal component (PCoA1) and second (PCoA2) explained 14.02% and 11.09% of the variation in microbial diversity, respectively. The samples in the Ctrl and Ca butyrate groups were tightly packed and not far apart, as shown in Fig. 4H. According to the taxonomic study, the structure of the cecal flora did not change following fermented Ca butyrate supplementation. At phylum levle, Bacteroidetes and Firmicutes were dominant in the cecal microbiota of hens. As compared with the Ctrl group, the diet with fermented Ca butyrate failed to change the top 7 most abundant bacteria (*P* > 0.05, Fig. 4I).

### 3.5 Inflammatory status

**Fig. 5:**
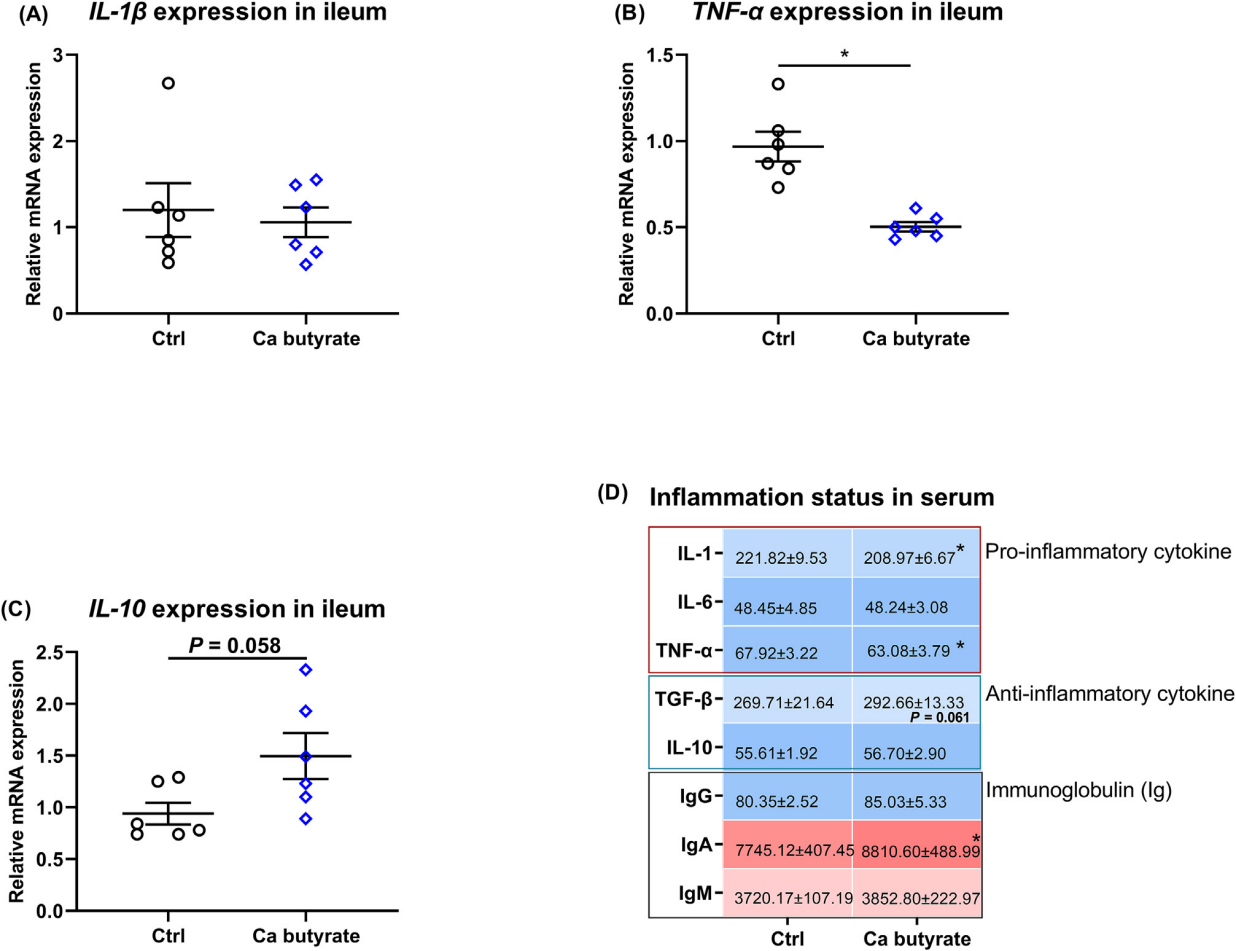
Diet with fermented calcium (Ca) butyrate depressed the inflammatory status of the ileum and serum in late-phase laying hens. The mRNA abundance of the pro-inflammatory factors (A) interleukin (*IL*)-1β and (B) tumor necrosis factor alpha (*TNF-α*), and the anti-inflammatory factors *IL-10* in the ileum. (D) Heatmap shows the concentration of the pro-inflammatory factors IL-1, IL-6, and TNF-α, the anti-inflammatory factors IL-10 and transforming growth factor beta (TGF-β), as well as immunoglobulins (Ig) G, IgA, and IgM in serum. Data shown are means and standard deviation (*n* = 6). ∗*P* < 0.05.

Based on the outcomes of the transcriptome, some parameters related to the inflammatory reaction in both ileum and serum were determined. As presented by Fig. 5, dietary fermented Ca butyrate treatment did not remarkably affect the mRNA level of ileal *IL-1β*, supplementation of 300 mg/kg fermented Ca butyrate to the diet resulted in a lower *TNF-α* mRNA level (*P* < 0.05), and an increased *IL-10* mRNA abundance (*P* = 0.058) in the ileum when compared to the Ctrl diet (Fig. 5A–C). Regarding the Ig, pro- and anti-inflammatory cytokines in serum, as presented in Fig. 5D, the decreased pro-inflammatory cytokine IL-1 and TNF-α, and elevated IgA were observed in laying hens fed Ca butyrate diet versus the Ctrl diet (both *P* < 0.05). The diet that included fermented Ca butyrate increased the anti-inflammatory cytokine TGF-β concentration relative to the Ctrl group (*P* = 0.061). There was no diet effect on serum IL-6, IL-10, IgG, and IgM content between Ctrl and Ca butyrate groups (*P* > 0.05).

### 3.6 Bone turnover and tibia quality

**Fig. 6:**
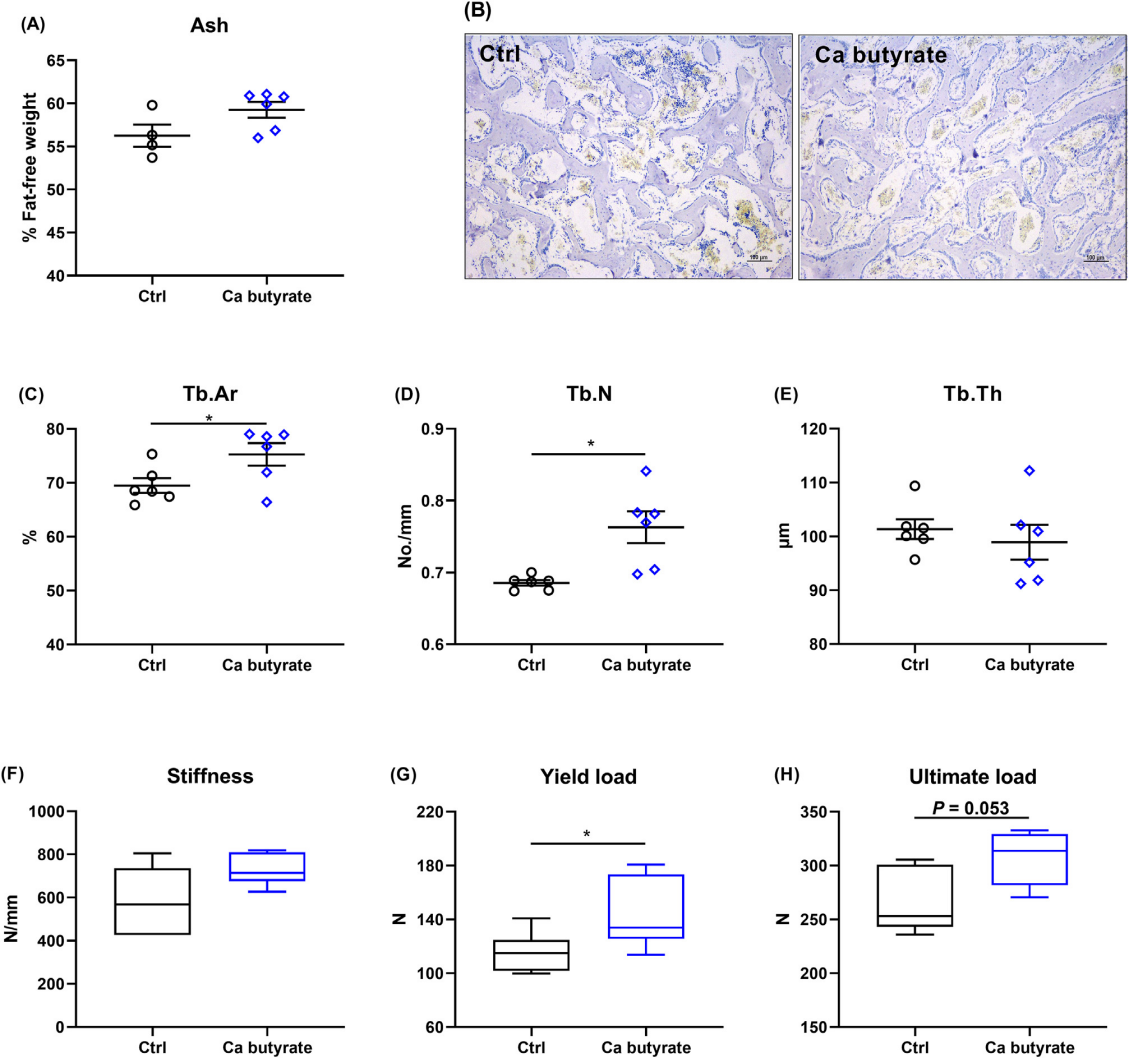
Dietary fermented calcium (Ca) butyrate supplementation improves microstructure and mechanical properties of tibia in laying hens. (A) Tibial ash, (B) toluidine blue stained (scale bar = 100 μm) and the morphometric analysis for (C) the trabecular bone area (Tb.Ar), (D) trabecular number (Tb.N), and (E) trabecular thickness (Tb.Th) determination by histomorphometry. Tibial mechanical properties including (F) stiffness, (G) yield load, and (H) ultimate load were measured. Boxes are bounded by the 25th and 75th percentiles, with the median shown by the line bisecting the box. Whiskers extend to the full range of the data. In the scatter plot, values are mean and standard deviation. ∗*P* < 0.05 and *n* = 6.

The effects of fermented Ca butyrate on bone growth, mineralization, and mechanical properties of tibia are presented in Fig. S2 and Fig. 6. The positive outcome for Ca butyrate was not associated with tibial growth, evidenced by similar tibial length, circumference, fresh weight, and relative weight (Fig. S2A-D). Dietary Ca butyrate inclusion also did not change Ca and P content (Fig. S2E), and tibial ash (Fig. 6A), as compared with the Ctrl group, whereas the Ca butyrate fed-birds exhibited higher Tb.Ar and Tb.N than the hens receiving the Ctrl diet, along with similar Tb.Th in the proximal tibia (Fig. 6B–E). Moreover, the mechanical testing analysis showed that the diet with fermented Ca butyrate significantly increased the yield load (*P* < 0.05) and tended to elevate the ultimate load (*P* = 0.053) of the tibia, but it did not affect the stiffness when compared to the Ctrl diet (Fig. 6F–H).

**Fig. 7:**
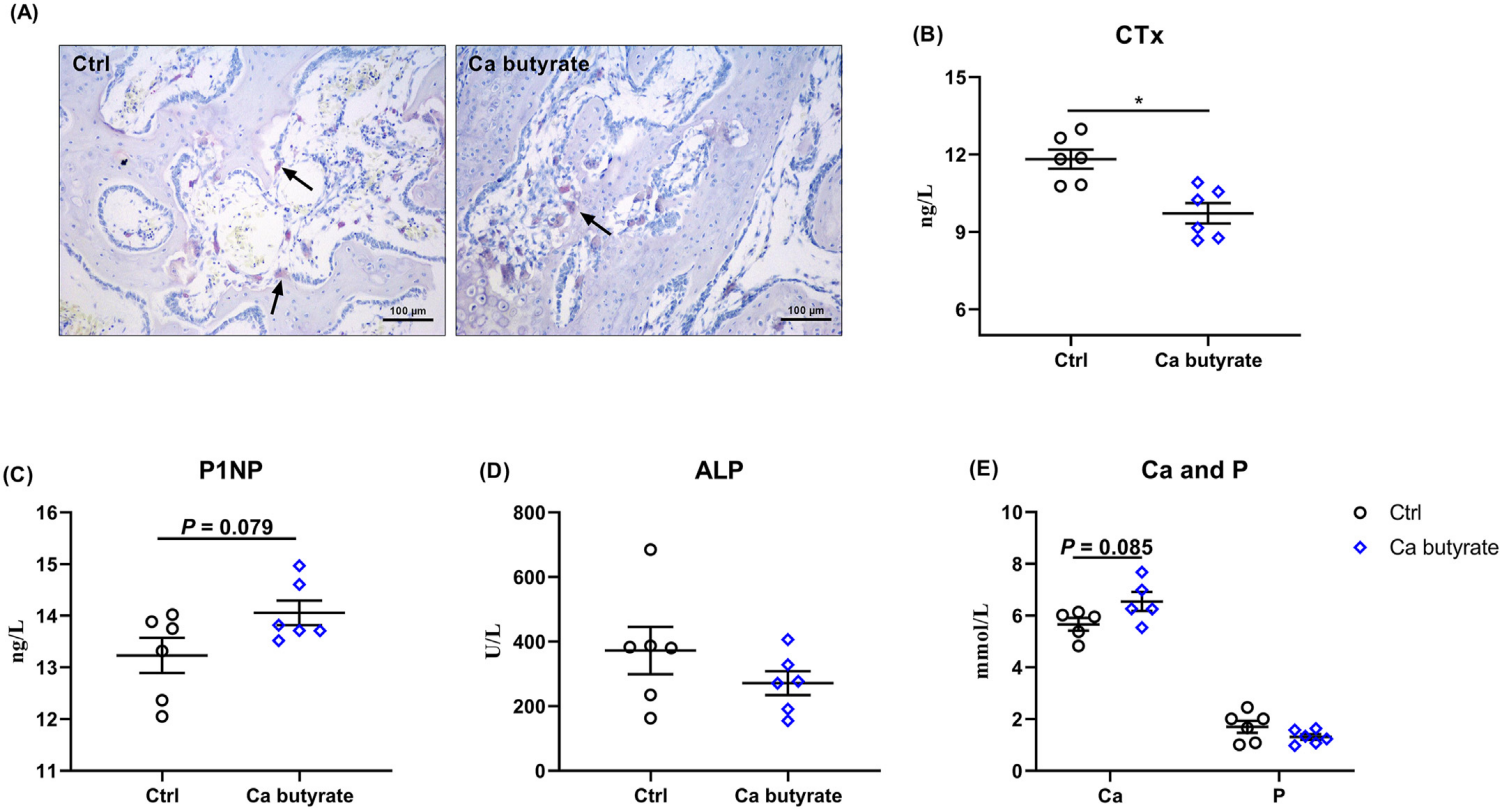
Impacts of dietary fermented calcium (Ca) butyrate on bone turnover of laying hens. (A) Representative tartrate-resistant acid phosphatase (TRAP) staining of tibia sections (scale bar = 100 μm), (B) bone resorption biomarker C-terminal cross-linked telopeptide of type I collagen (CTx) content, and bone formation biomarkers including (C) procollagen type I intact N-terminal propeptide (P1NP) concentration and (D) alkaline phosphatase (ALP) activity were determined. (E) Serum Ca and phosphorous (P) contents. Data shown are means and standard deviation (*n* = 6). ∗*P* < 0.05.

Regarding the bone turnover, the tibia was stained using TRAP and showed that TRAP-positive cells from Ca butyrate birds were lower in the proximal tibia sections than the Ctrl groups (Fig. 7A). The serum level of CTx, which reflects bone resorption, was decreased in Ca butyrate compared with Ctrl birds (*P* < 0.05; Fig. 7B). In regard to serum bone formation markers, Ca, and P concentration in serum, the addition of fermented Ca butyrate increased the level of P1NP (*P* = 0.079) and Ca (*P* = 0.085) when compared to Ctrl group. However, no obvious differences in the serum ALP activity and P content were observed (Fig. 7C–E).

## 4 Discussion

Extending the egg production period might result in poor production performance, inferior egg quality, and increased mobility problems accompanied by decreased immune capacity and intestinal dysfunction, resulting in substantial economic losses and impaired animal welfare for laying hens (Elhamouly et al., 2018a; Samiullah et al., 2017). Outcomes from the present results suggested that dietary fermented Ca butyrate supplementation could improve ovarian function by increasing egg weight and the number of ovarian follicles, which might be associated with changes in ovarian immune status. Moreover, the dietary inclusion of fermented Ca butyrate was associated with the enhanced intestinal barrier and depressed systemic inflammation, contributing to an improvement in tibial microstructure and mechanical properties through modifying bone turnover in post-peak laying hens.

Both laying rate and egg quality are the most important indicators of the condition of laying hens, yet the effects of butyrate on laying performance are inconsistent. Some studies suggested that dietary supplementation of sodium butyrate or Ca butyrate did not change egg production in hens (Song et al., 2022; Zhang et al., 2022c), whereas the supplementation with sodium butyrate (0, 300, 500, and 800 mg/kg) could increase egg weight in a dose-dependent manner from age 51 to 62 weeks (Zhang et al., 2022c). In this study, the dietary Ca butyrate treatment (300 mg/kg) was found to increase egg weight. It was reported that the increase in egg weight might derive from a reduced feed intake (Li et al., 2011) and/or a heavier body weight (Renden et al., 1984). However, no significant changes in both body weight and feed intake during the trial suggested that the increased egg weight due to dietary fermented Ca butyrate supplementation seldom bears any relationship to feed consumption or body weight. Of note, the diet with 0.5% Ca butyrate was noticed to significantly reduce the feed intake of 70-week-old Isa Brown hens (Song et al., 2022). The comparable feed intake and feed efficiency between Ctrl and Ca butyrate groups from 50 to 58 weeks of age indicated that laying performance is not deteriorated by fermented Ca butyrate and further study is needed to illustrate this relationship. As an additive, Ca butyrate might eventually decompose to produce butyric acid, and the latter was confirmed to promote the egg quality of laying hens including eggshell strength in 48 weeks of age Jinghong-1 strain laying hens (Zhan et al., 2019). Accordingly, the current study revealed that dietary fermented Ca butyrate supplementation tended to increase the strength of eggshells. Collectively, these results suggest that dietary supplementation of 300 mg/kg fermented Ca butyrate could improve egg weight and tended to increase the eggshell strength of post-peak laying hens.

It is well-established that egg quality is impacted by multiple factors, including diet and age of laying hens (Kowalska et al., 2021). Aging and the associated dysfunction of the ovary may be the main reason for the decline in rate of egg production, especially the depleted follicle pool during the later laying period (Faddy, 2000). In the present study, the number of ovarian follicles were improved by the treatment of fermented Ca butyrate. This might be attributed to the increased secretion of follicle-stimulating hormone due to butyrate administration, subsequently stimulating the growth and development of follicles (Ghosh and Cox, 1977). The precise mechanism underlying the regulatory mechanism of butyrate on the development of ovarian follicles is still unknown. As one of the important characteristics of oocytes, transcriptomic profiling of granulosa may help identify biomarkers of the oocyte and find the relationship between butyrate and the development of ovarian follicles. The results of using RNA-seq in this study showed that 186 up-regulated genes and 67 downregulated genes at the translational level were identified in the Ca butyrate group when compared to the Ctrl group. In addition to purine and tyrosine metabolism, a significant enrichment in the immune pathway was also noticed, including cytokine and TLR signaling pathways. Indeed, it was reported that TLRs could be expressed in ovarian follicles and that the expressions of TLR4 and TLR5 were increased with the growth of follicles (Subedi et al., 2007). More importantly, age could affect the expression of inflammatory cytokines in the uterine mucosa of laying hens (Elhamouly et al., 2018a), in which the number of macrophages tended to increase with age in the oviduct (Zheng and Yoshimura, 1999). A study on mice found that ovarian aging characterized by decline in follicle number and oocyte quality was associated with increased levels in intra-ovarian CD4^+^ T cells, B cells, macrophages, as well as several cytokines and chemokines (Lliberos et al., 2021), indicating that cytokine secretion plays a critical role in the ovary, as some of these cytokines and TNF-α in particular, are known to induce ovarian follicle death. From this point, attenuating the ovarian inflammation mediated by the immune system in the late stage of the production period might account for the increase in the ovarian follicles in this study (Elhamouly et al., 2018b; Samiullah et al., 2017).

Taking into consideration how dietary fermented Ca butyrate relieve the inflammation of the ovary to improve egg quality, one of the potential mechanisms is to promote intestinal barrier function and decrease the systemic inflammatory response. It is known that the intestine plays a crucial role in preventing the passage of pathogens, antigens, and bacterial toxins into the systemic circulation. The relationships between intestinal integrity in laying performance and egg quality have been established (Abbasi et al., 2021; Gan et al., 2020; Zhang et al., 2022c). It was reported that weakened intestinal mucosal barrier function probably induces an increase in inflammation, inhibits ovarian follicular growth, and causes a decrease in laying performance (Nii et al., 2020). Evidence exists that points out that butyric acid exerts beneficial effects on intestinal integrity since it could serve as the primary source of energy for enterocytes and epithelial maintenance (Abdelqader and Al-Fataftah, 2016; Celasco et al., 2014; Elnesr et al., 2019). Similar to the positive roles of sodium butyrate in the intestinal health of laying hens (Wang et al., 2021; Zhang et al., 2022c), the diet supplemented with 0.1% Ca butyrate significantly increased the villus height of the duodenum in broilers, and dietary Ca butyrate supplementation (0, 0.3, 0.5, and 0.7 g/kg diet) dose-dependently increased the villus height and decreased the crypt depth of the duodenum in Japanese quails. In the current study, the diet with 300 mg/kg fermented Ca butyrate facilitated ileal integrity, evidenced by increased villus height, and upregulated mRNA levels of occludin and claudin-1. Of note, the beneficial roles performed by Ca butyrate in the ileum were probably related to its less soluble form, which allows Ca butyrate to dissolve slowly, and butyric acid is released gradually along the intestinal tract. It is analogous to the increase in ileal villus height due to dietary-coated sodium butyrate treatment in 51-week-old laying hens (Zhang et al., 2022c). In addition, relationships between butyrate and gut microbiota were found in layers. For instance, dietary sodium butyrate administration (2.5 and 5 g/kg) reduced total bacteria and ileal *Escherichia coli* (*E. coli*) numbers in 55-week-old hens (Jahanian and Golshadi, 2015). With the increase in dietary sodium butyrate levels (0, 300, 500, and 800 mg/kg), the abundances of Bacteroidota, Firmicutes, and Deferribacteres were increased quadratically in cecal contents of laying hens (Zhang et al., 2022c). Both *E. coli* and *Clostridium perfringens* numbers in the excreta were reduced in these quails fed Ca butyrate diets in a dose-dependent manner (Abd El-Wahab et al., 2019). However, in this study, dietary fermented Ca butyrate supplementation did not significantly alter the diversity and composition of cecal microbiota according to the results of 16S rRNA sequencing, which is in agreement with the previous study saying that the abundance of cecal bacterial count in broilers was comparable between Ca butyrate and control diets (Pineda-Quiroga et al., 2017). Even so, as a consequence of enhancement of the intestinal barrier, the downregulated expression of pro-inflammatory cytokine *TNF-α* and upregulated level of anti-inflammatory *IL-10*, albeit not significantly, were observed in hens receiving a fermented Ca butyrate diet in the present study, implying that the supplementation of fermented Ca butyrate depressed the inflammatory response in the intestine. A study on piglets has demonstrated that 90% Ca-butyrate combined with 10% tannin extract supplementation could reduce the level of TNF-α in the duodenum (Maito et al., 2022). Reflecting on serum, the treatment of Ca butyrate reduced the contents of IL-1, and TNF-α in serum, while it tended to increase the level of TGF-β in the current study, which supports the recent findings that Ca butyrate supplementation at 0.7 g/kg reduced the levels of TNF-α, IL-6, and IL1-β, but increased the concentration of serum IL-10 in Japanese quails (Abd El-Wahab et al., 2019). In addition, increased levels of serum IgA, a key immune mediator, were also observed in Ca butyrate-fed hens. Previous studies have suggested that increased IgA is linked to improving the immune function of birds (Zhang et al., 2022a). Altogether, these findings indicated that fermented Ca butyrate decreased the pro-inflammatory cytokines in the ileum and serum and increased serum IgA levels to strengthen intestinal and systemic immune function, likely relieving inflammation of the ovaries. In this regard, alterations to the transcription profile of granulosa were also identified as positive effects of fermented Ca butyrate on ovarian function that are connected with cytokines, TLR signaling pathways and the intestinal immune network, especially IgA production.

In addition to reducing ovarian inflammation, optimizing bone quality might be one of the alternatives for improving egg production and quality in post-peak layer hens on account of Ca storage for eggshell formation. During egg formation in laying hens, large numbers of active osteoclasts are recruited to resorb and mobilize Ca from the medullary bone to meet the requirement of eggshell formation. With the extended laying cycle, the bone quality might gradually decrease as a result of insufficient bone formation and excessive bone resorption (Dacke et al., 1993), resulting in a higher incidence of bone fractures in furnished cages (Rodenburg et al., 2008; Wei et al., 2021). In the present study, dietary fermented Ca butyrate did not change tibial growth and mass, as evidenced by similar dimension, weight, and mineral content, whereas the fermented Ca butyrate increased calcified area, trabecular number, yield load, and ultimate load, which indicated an improvement in tibial microstructure and mechanical properties. Similarly, obesity-prone rats treated with 4% sodium butyrate also proved that butyrate could preserve bone metabolism by enhancing femur load and bone mineral density (Tang et al., 2020). However, studies on laying hens found that a diet supplemented with 0.5% Ca butyrate had no significant effect on tibial strength, but it decreased the Ca and P levels of both bone and serum, along with increased eggshell strength and thickness (Song et al., 2022), which is in contrast to our findings. Possible explanations for the discrepancy in results regarding tibial mass and strength might be attributed to eggshell properties and systemic Ca–P homeostasis. In this study, dietary supplementation of 300 mg/kg fermented Ca butyrate tended to increase eggshell strength but not eggshell thickness of post-peak laying hens. Combined with similar Ca and P concentrations in serum, it is possible that sufficient Ca and P were absorbed to form the eggshell therefore avoiding mobilization of bone Ca and P, leaving them available to contribute to the improvement of tibia quality in post-peak laying hens.

To understand the mechanism of dietary fermented Ca butyrate on bone quality, bone remodeling was further evaluated in this study. Published data showed that impaired intestinal integrity challenged with *Salmonella* infection (Raehtz et al., 2018) or heat stress (Zhang et al., 2021) decreased the bone mass of chickens, which was accompanied by systemic inflammation and bone resorption. Decreased the secretion of inflammatory cytokines through enhancing the intestinal barrier could restore bone loss in broilers (Zhang et al., 2021, 2022a). These observations suggest the importance of the interaction between the intestinal barrier and inflammation in the bone metabolism of domestic birds (D'Amelio and Sassi, 2018; Sjögren et al., 2012; Zhang et al., 2021), and bone resorption mediated by inflammatory cytokine might be a potential inducer for inferior bone quality. Several studies reported that the proinflammatory cytokine especially TNF-α and IL-1 contributed to osteoclastogenesis through interaction with nuclear factor kappa B receptor activating factor ligand (RANKL) in osteoclast precursor cells (Xing et al., 2005), and promoted the expression of osteoclast specific genes such as cathepsin K and TRAP (Chatziravdeli et al., 2019; Xing et al., 2005). Anti-TNF-α therapy could reverse the increase in osteoclast precursors in the circulation of mice suffering from arthritis (Li et al., 2004). Because Ca butyrate has been found to exert notable inflammatory effects in vivo and in vitro (Celasco et al., 2014), one might expect it to inhibit bone resorption. In the present study, the diet with fermented Ca butyrate reduced the number of TRAP-positive cells in bone sections and the circulatory level of CTx, a marker of bone resorption, suggesting that dietary Ca butyrate supplementation downregulates bone resorption. This was supported by a previous study, where dietary sodium butyrate intervention inhibited the expression of genes related to osteoclast differentiation, including cathepsin K in obesity-prone rats (Tang et al., 2020). Besides this, the reduction of osteoclast formation and resorption activity might be a direct role of butyrate. G protein-coupled receptor (GPR)-43 is a well-known receptor for butyrate and is required for the inhibitory effects of short-chain fatty acids on osteoclast (Montalvany-Antonucci et al., 2019). Stimulation of GPR43 with a selective agonist led to a significant reduction in osteoclast formation in mice (Wallimann et al., 2021). Therefore, it is possible that Ca butyrate directly combined with GPR43 to prevent osteoclast formation in the current study, however this requires further verification. Taken together, Ca butyrate-mediated inhibition of osteoclast formation is a likely consequence of effects on multiple pathways including immune-related and/or GPR signaling in late-phase laying hens.

Regarding the relationship between butyrate and bone formation, the effects of sodium butyrate on the differentiation of osteoblasts, the main cells that mediate bone formation, were examined in previous studies (Katono et al., 2008; Lee et al., 2006; Zhao et al., 2007). Research on human osteoblasts found that sodium butyrate (0, 10^−8^, 10^−6^ or 10^−4^ mol/L) stimulated bone formation in a dose-dependent manner by facilitating mineralized nodule formation and the Ca content of mineralized nodules (Katono et al., 2008). In this study, dietary supplementation of fermented Ca butyrate tended to increase the level of P1NP, a bone formation marker, implying that fermented Ca butyrate treatment might have a potential role in promoting bone formation in laying hens. ALP plays a key role in the calcification of bone via hydrolyzing the ester bond of organic phosphate compounds under alkaline conditions. Published data demonstrated that sodium butyrate could accelerate osteogenesis by inducing the expression of osteoblast markers including ALP (Lee et al., 2006). However, in the present study, ALP activity was not affected by the addition of fermented Ca butyrate. This suggests that the growth of hydroxyapatite crystals induced at the initiation of mineralization by ALP may not be related to the presence of Ca butyrate. Still, more studies about the effects of fermented Ca butyrate on bone formation and the mechanism underlying Ca butyrate interference with the activity of osteoblasts are needed in laying hens.

Finally, further study is still required to elaborate on the following questions. The first is the limitation of crude protein and butyrate assessment. The use of ileum content but not feces will be more accurate to indicate the protein digestibility, and the determination of butyrate in diet and feces might help to discover the effect of butyrate on the laying production, ovarian function, and tibia quality for post-peak laying hens. The second is experimental design. It is well-known that dietary fermented Ca butyrate supplementation can increase the Ca level of diets, thereby affecting the tibia quality and egg quality, which did not elaborate based on our current outcomes. In addition, the contributions of *C. butyricum* included in fermented Ca butyrate to ovarian function, intestinal integrity, and tibia quality of hens cannot be ruled out, and thus a *C. butyricum* group is needed in this experimental design. The third is radiological assessment. Due to our insufficient skills for the micro-CT analysis, the morphology of the tibia was evaluated using toluidine blue staining. Therefore, we admit the possibility that some of our conclusions may include overestimation or underestimation of the role of fermented Ca butyrate in the bone metabolism of post-peak laying hens.

## 5 Conclusions

In summary, the results of this study indicated that supplementation of 300 mg/kg fermented Ca butyrate in late-phase laying hen diets positively affected egg weight and the number of ovarian follicles during an 8-week laying period, which might be associated with changes in ovarian immune function. In addition, improvements in tibial microstructure and mechanical properties through decreasing bone resorption were also observed when adding fermented Ca butyrate to diets. This was closely related to the enhancement of the intestinal barrier and the depression in systemic inflammation.

## Author contributions

**Huaiyong Zhang:** conceptualization, methodology, sampling, experimentation, data curation, funding acquisition, writing the original draft, and editing. **Yongshuai Wang:** methodology, sampling, experimentation, preliminary analyses, writing the original draft and editing. **Bin Wei:** methodology, sampling, and experimentation. **Yilu Wang:** sampling and experimentation. **Leilei Wang:** methodology and sampling. **Minh Tu Nguyen:** software, advanced analyses, and graph-making. **Xiangyun Lv:** project administration and methodology. **Yanqun Huang:** experimentation and review. **Wen Chen:** project administration, funding acquisition software, and writing-review and editing.

## Declaration of competing interest

We declare that we have no financial and personal relationships with other people or organizations that can inappropriately influence our work, and there is no professional or other personal interest of any nature or kind in any product, service and/or company that could be construed as influencing the content of this paper.

## Availability of data and materials

Sequences generated for microbiome have been deposited in the NCBI database under the accession number PRJNA949874, and the sequencing files of RNA-seq were deposited to the BioProject database in NCBI (accession numbers PRJNA947743). Other data will provide for reasonable requests.
