# Supplementary material for: Fermented calcium butyrate supplementation in post-peak laying hens improved ovarian function and tibia quality through the “gut-bone” axis
Source: Anim Nutr. 2024 Jan 3;16:350–62. doi: 10.1016/j.aninu.2023.10.008 (PMC10867563; doi:10.1016/j.aninu.2023.10.008)
Supplement: Multimedia component 1 [file mmc1.docx]

**Table S1.** Ingredients and nutrient composition of the basal diet (as-fed basis)

| **Ingredients** | **Proportion, %** | **Calculated analysis** | **Proportion, %** |
| --- | --- | --- | --- |
| Corn | 32.95 | AME, MJ/kg | 10.96 |
| Wheat | 30.00 | CP | 16.30 |
| Soybean meal (46% CP) | 12.01 | Calcium | 3.60 |
| Wheat bran | 3.70 | Total phosphorus | 0.52 |
| Shotcrete corn husk | 2.50 | Available phosphorus | 0.30 |
| Corn gluten powder | 1.50 | Lysine | 0.88 |
| DDGS | 3.50 | Methionine + cystine | 0.67 |
| Sodium chloride | 0.24 | Threonine | 0.61 |
| Limestone | 8.60 |  |  |
| CaHPO_4_ | 0.43 |  |  |
| Chicken bone meal | 2.00 |  |  |
| Soybean oil | 0.57 |  |  |
| Premix ^1^ | 2.00 |  |  |
| Total | 100.00 |  |  |

DDGS = distillers dried grains with soluble; AME = apparent metabolism energy; CP = crude protein.

^1^ Provided per kilogram of diet: Cu (CuSO_4_·5H_2_O), 8 mg; Fe (FeSO_4_·7H_2_O), 80 mg; Zn (ZnSO_4_·7H_2_O), 80 mg; Mn (MnSO_4_·H_2_O), 80 mg; Se (NaSeO_3_), 0.3 mg; I (KI), 0.7 mg; vitamin A, 12,700 IU; vitamin D, 5,000 IU; vitamin E, 70 IU; vitamin K, 5.0 mg; thiamine, 37.0 mg; riboflavin, 12.0 mg; pyridoxine, 3.0 mg; vitamin B_12_, 0.7 mg; calcium pantothenate, 10.0 mg; folate, 0.5 mg; biotin, 4 mg; nicotinic acid 80 mg; choline chloride, 100 g.

**Table S2.** Analysis of diet composition (as-fed basis)

| **Items** | **Ctrl diet** | **Fermented Ca butyrate diet** |
| --- | --- | --- |
| Dry matter, % | 90.15 ± 3.38 | 89.23 ± 2.73 |
| Apparent metabolizable energy, kcal/kg | 2634.73 ± 10.42 | 2644.82 ± 16.40 |
| Crude protein, % | 17.77 ± 1.33 | 17.42 ± 1.58 |
| Calcium, % | 3.51 ± 0.11 | 3.66 ± 0.87 |
| Total phosphorus, % | 0.48 ± 0.01 | 0.46 ± 0.01 |

**Table S3.** The RT-PCR primers

| **Gene ID** | **Gene** | **Primer sequences (5’→3’)** | **Product length, bp** |
| --- | --- | --- | --- |
| XM_046925214.1 | *ZO-1* | F: GAAGAGAGCACAGAACGCAG | 123 |
|  |  | R: CACTTGTGGCAAGCTGAAGT |  |
| NM_001013611.2 | Claudin-1 | F: TCTGGTGTTAACGGGTGTGA | 117 |
|  |  | R: GTCTTTGGTGGCGTGATCTT |  |
| NM_205128.1 | Occludin | F: CGTTCTTCACCCACTCCTCC | 107 |
|  |  | R: CCAGAAGACGCGCAGTAAGA |  |
| NM_001039258.3 | *CDH1* | F: AGCCAAGGGCCTGGATTATG | 157 |
|  |  | R: GATAGGGGGCACGAAGACAG |  |
| NM_001318434.1 | *MUC2* | F: AGTGGCCATGGTTTCTTGTC | 80 |
|  |  | R: TGCCAGCCTTTTTATGCTCT |  |
| XM_025148915.2 | *ALK* | F: GCTCCAGGAACAAGCCTACC | 126 |
|  |  | R: CGTGTCCCAAGTTCTCCCTC |  |
| NM_001201386.2 | *OC3* | F: AAGCTACCCTCCCTGTGACT | 138 |
|  |  | R: AAGATTCTTTCTGCCGGGGG |  |
| XM_046927920.1 | *MYO15A* | F: GGGGACCCCTATGAGGACTT | 93 |
|  |  | R: GCTGTACTGGATGTGGTGCT |  |
| NM_204805.2 | *TH* | F: TCACGAGCGCTTAAGGTGTT | 95 |
|  |  | R: AGTTCAGCAGTCCCTTCACG |  |
| NM_204259.2 | *PTGDS* | F: GGGTGAACAGTGCGAGAAGA  R: ACACGGATGTCATGGTTGCT | 105 |
| NM_205518.1 | β-Actin | F: GTCCACCGCAAATGCTTCTAA | 78 |
|  |  | R: TGCGCATTTATGGGTTTTGTT |  |

*ZO-1* = zonula occludens-1; *CDH1* = cadherin 1; *MUC2* = mucin-2, oligomeric mucus/gel-forming; *ALK* = anaplastic lymphoma kinase; *OC3* = osteocalcin-like protein OC3; *MYO15A* = myosin XVA; *PTGDS* = prostaglandin D2 synthase; *TH* = tyrosine hydroxylase.
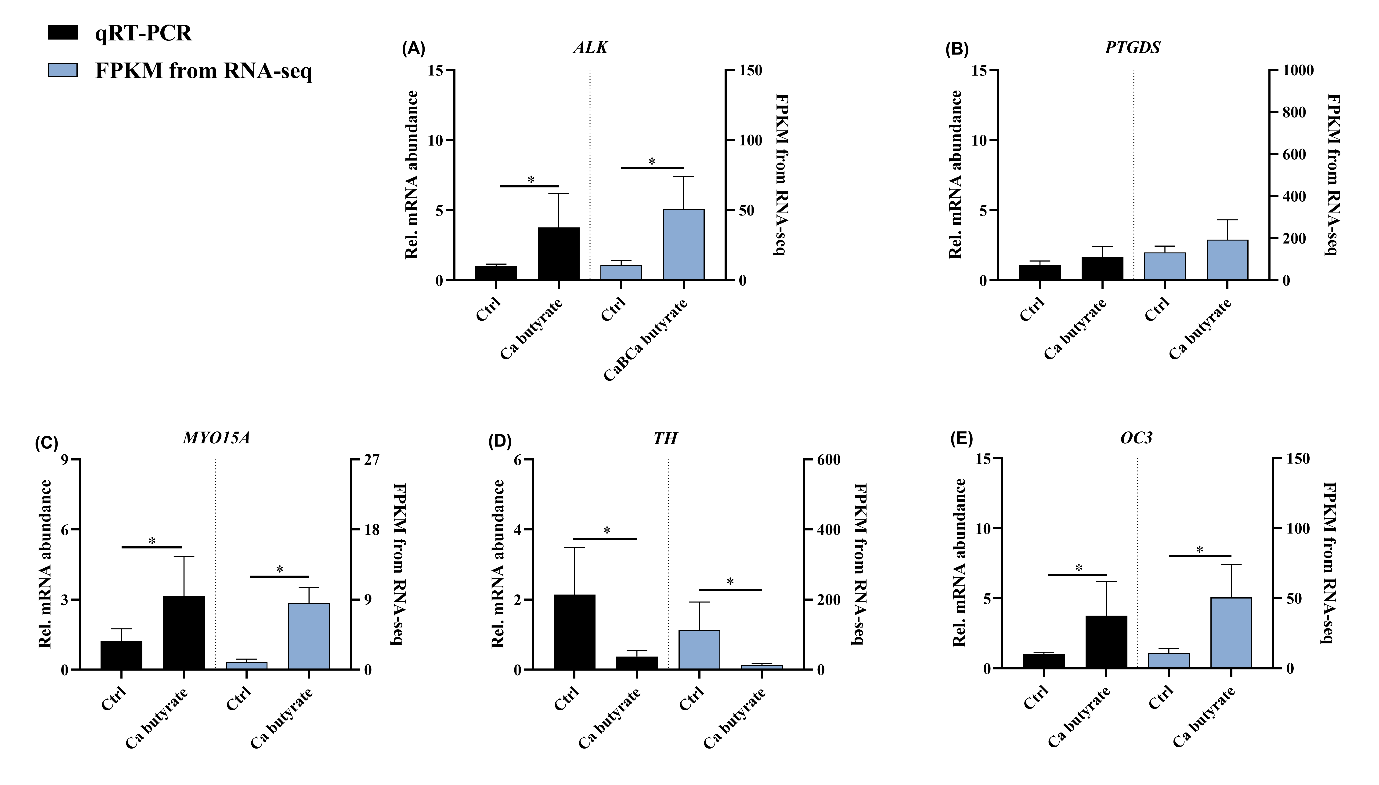


**Fig. S1**. Verification of transcriptome analysis (RNA-seq) and RT-PCR using anaplastic lymphoma kinase (*ALK*), prostaglandin D2 synthase (*PTGDS*), myosin XVA (*MYO15A*), tyrosine hydroxylase (*TH*), and osteocalcin-like protein OC3 (*OC3*). Data shown are means and standard deviation (*n* = 6). ^∗^*P* < 0.05.


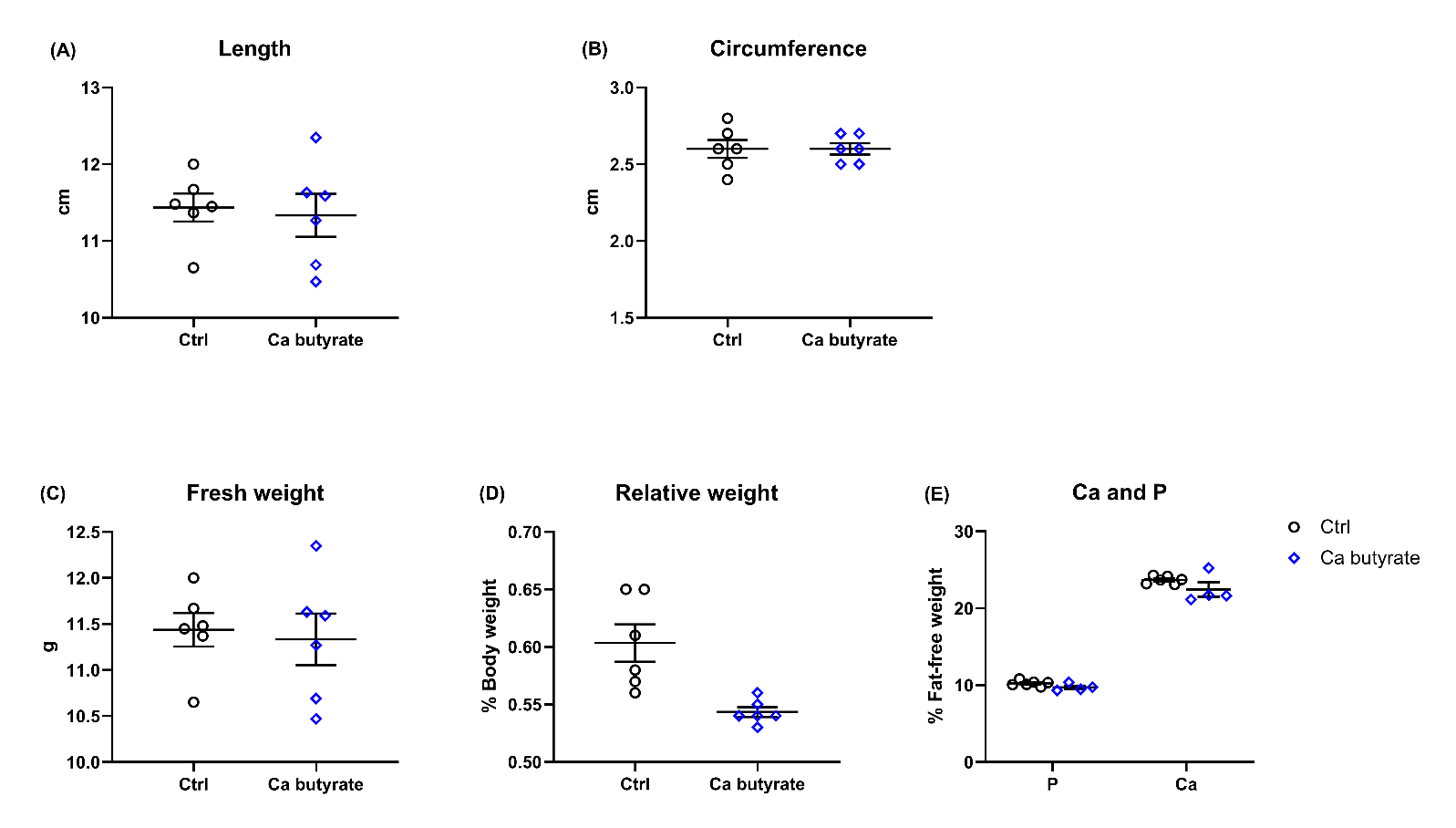


**Fig. S2.** Effect of fermented calcium (Ca) butyrate supplementation on the tibia growth showed by (A) length, (B) circumference, (C) fresh weight, and (D) relative weight, and (E) Ca and phosphorus (P) content of tibia. Data shown are means and standard deviation (*n* = 6).
